# Simultaneous editing of *CsLOB1* and *CsWRKY22* in citrus confers resistance to citrus canker

**DOI:** 10.1093/hr/uhag137

**Published:** 2026-04-10

**Authors:** Mengyuan Hou, Rongrong Qu, Mengting He, Xiangling Chen, Lanzhen Xu, Juanjuan Ma, Tiangang Lei, Yongrui He, Xiuping Zou

**Affiliations:** Citrus Research Institute, Southwest University, Chongqing 400712, China; Citrus Research Institute, Southwest University, Chongqing 400712, China; Citrus Research Institute, Southwest University, Chongqing 400712, China; Horticultural Research Institute, Guangxi Academy of Agricultural Sciences, Nanning 530007, China; Citrus Research Institute, Southwest University, Chongqing 400712, China; Citrus Research Institute, Southwest University, Chongqing 400712, China; Citrus Research Institute, Southwest University, Chongqing 400712, China; Citrus Research Institute, Southwest University, Chongqing 400712, China; Citrus Research Institute, Southwest University, Chongqing 400712, China

Dear Editor,

Citrus serves as a rich source of essential nutrients and bioactive compounds, including vitamins, minerals, and dietary fiber, and is the most widely produced fruit globally. However, growers face many biotic and abiotic challenges, including citrus canker induced by *Xanthomonas citri* subsp. *citri* (*Xcc*), which has caused significant economic losses to the global citrus industry [1]. Editing disease susceptibility genes is a promising approach to improve resistance to citrus canker [2]. Further, multiplex gene editing has shown great potential for enhancing durable resistance in plants. However, limited information is available regarding the simultaneous modification of multiple genes to confer disease resistance in citrus.

*Xcc* secretes effector proteins into host cells to successfully infect citrus plants. These secreted molecules reprogram cellular processes, ultimately inducing the characteristic symptoms of citrus canker, including chlorotic or water-soaked halos, hyperplastic spots, volcanic-like lesions, and brown corky cankers [1, 3]. The cellular development of these symptoms is marked by host hyperplasia (excessive cell division) and hypertrophy (cell enlargement) [1]. The pathogenicity A (PthA) 4 effector of *Xcc* binds to the effector-binding element (EBE_PthA4_) in the promoter of *Citrus lateral organ boundaries 1* (*CsLOB1*) to upregulate *CsLOB1* expression, triggering bacterial growth and host symptom development [4]. Previous studies (Fig. 1a) revealed a central role of *CsLOB1* in hyperplasia and canker induced by *Xcc* [1], whereas *CsWRKY22* promoted susceptibility to citrus canker by inducing host hypertrophy [3]. Further, CsWRKY22 upregulated *CsLOB1* expression by directly binding to the *CsLOB1* promoter (Fig. 1a). Editing *CsLOB1* and *CsWRKY22* conferred resistance to citrus canker [2, 5]. These discoveries prompted the simultaneous mutation of *CsWRKY22* and *CsLOB1* to enhance resistance to citrus canker.

**Figure 1:**
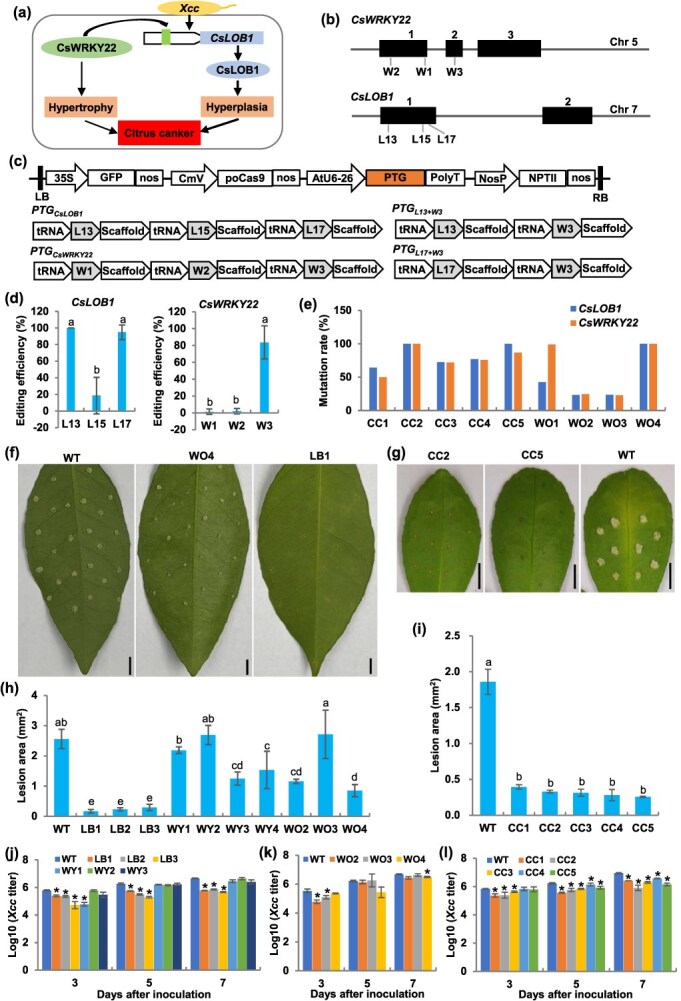
Double editing of *CsLOB1* and *CsWRKY22* for resistance to citrus canker caused by *Xcc*. (a) Synergistic mechanism of CsWRKY22 and CsLOB1 in regulating susceptibility to citrus canker. (b) *CsWRKY22* and *CsLOB1* structures and the positions of the gRNAs. Exon positions are marked with black rectangles. (c) Structures of the vector and the *PTGs* for editing *CsLOB1* and *CsWRKY22*. *PTG_CsLOB1_* contained L13, L15, and L17 gRNA spacers for editing *CsLOB1*, whereas *PTG_CsWRKY22_* contained W1, W2, and W3 gRNA spacers for editing *CsWRKY22*. *PTG*_*L*13*+W*3_ and *PTG*_*L*17*+W*3_ contained L13 or L17 and W3 gRNA spacers for the simultaneous editing of *CsLOB1* and *CsWRKY22*. (d) Editing activity induced by various gRNA spacers in Wanjincheng orange. Value is expressed by the mean ± standard deviation of three to four independent transgenic plants (Figs S4 and S5). Different letters on the bars denote significant differences (Duncan’s test, *P* < 0.05). (e) Mutation rate of *CsLOB1* and *CsWRKY22* in double-edited plants. Results are supported by Figs S7 and S8. (f–i) Citrus canker lesions (f and g) and lesion size (h and i) on leaves at 7 dpi. Scale bars = 0.5 cm. (j–l) *Xcc* growth (log10 of *Xcc* titer) in leaves. Based on the copy number of the *Xcc pthA* gene in leaves, *Xcc* titer was quantified using qPCR. ^*^ and different letters represent significant difference compared with WT control (Duncan’s test, *P* < 0.05). LB# and WY#: *CsLOB1* and *CsWRKY22* Wanjincheng orange mutants, respectively; WO# and CC#: double mutants of Wanjincheng orange and Carrizo citrange mutants, respectively.

In this study, a polycistronic *tRNA:gRNA* (*PTG*) gene was used to guide *CRISPR*/Cas9-mediated simultaneous editing of *CsLOB1* and *CsWRKY22*. To this end, two plasmids, pGST and pATS, were constructed (Fig. S1a and Table S1). pGST contained a gRNA:tRNA fusion, which served as a template to synthesize the cistronic unit of *PTG* using polymerase chain reaction (PCR). pATS contained an expression cassette comprising the AtU6–26::tRNA:sgRNA scaffold–pol III terminator, which was used to construct the expression cassette of *PTG* driven by the *Arabidopsis* AtU6–26 promoter. tRNA was a 77-bp-long pre-tRNA^Gly^ fragment (Table S1). A strategy to synthesize *PTG* by integrating PCR and restriction digestion is shown in Fig. S1b. In brief, gRNA spacers were added to the ends of the gRNA scaffold:tRNA platforms using PCR. Then, the gRNA scaffold:tRNA platforms were ligated together through the 4-bp overhanded sequences generated using *Bbs*I to produce *PTG* with various gRNA spacers. The *PTG* was further amplified from the ligated product and cloned into the pATS vector through *Bbs*I digestion to generate the *PTG* expression cassette.

Seven gRNA spacers (Fig. 1b and Fig. S2a and Table S2) targeting *CsLOB1* were selected via *in vitro* cleavage analysis, with L11, L12, L13, L15, L16, and L17 demonstrating higher cleavage activity (Fig. S2b). L11 bound to a sequence in the *CsLOB1* promoter located far from the EBE_PthA4_ site (Fig. S3). This distance made it difficult to delete the EBE_PthA4_ sequence, an event crucial for conferring high resistance to citrus canker [2]. Additionally, the target sequences of L12 and L16 contained single-nucleotide polymorphisms (Fig. S3), which hindered the efficient modification of the two variable alleles of *CsLOB1* [5]. Therefore, L13, L15, and L17 were selected to construct the *PTG* to edit *CsLOB1*. The previously reported W1, W2, and W3 gRNA spacers (Figs 1b and S3) [5] were employed for editing *CsWRKY22*. First, *PTG_CsLOB1_* and *PTG_CsWRKY22_* were constructed to separately edit *CsLOB1* and *CsWRKY22* (Fig. 1c). The constructs were introduced into Wanjincheng orange (*Citrus sinensis* Osbeck) via *Agrobacterium*-mediated transformation [2]. Three *PTG_CsLOB1_* (LB1–3 lines) and four *PTG_CsWRKY22_* (WY1–4 lines) transgenic plants were identified (Fig. S4a-c). Sanger sequencing revealed that L13 (100%), L17 (84.6%–100%), and W3 (60.61%–100%) gRNA spacers had high editing activities (Figs 1d, S5, and  S6). LB1, LB2, and LB3 transgenic lines and WY3 and WY4 transgenic lines had 100% *CsLOB1* and *CsWRKY22* mutations, respectively (Figs S5 and S6).

Based on the aforementioned results, L13, L17, and W3 gRNA spacers were selected to construct *PTG* genes for simultaneously editing *CsLOB1* and *CsWRKY22*. *PTG*_*L*13*+W*3_ contained L13 and W3, whereas *PTG*_*L*17*+W*3_ had L17 and W3 (Fig. 1c). These resulting constructs were introduced into Wanjincheng orange and Carrizo citrange (*Poncirus trifoliata* (L.) Raf. *× Citrus sinensis* Osbeck) to generate transgenic plants. Five transgenic Carrizo citranges (CC1–5 lines) with *PTG*_*L*17*+W*3_, three transgenic Wanjincheng oranges (WO1–3 lines) with *PTG*_*L*17*+W*3_, and one transgenic Wanjincheng orange (WO4) with *PTG*_*L*13*+W*3_ were obtained (Fig. Sa7 and b). All the transgenic plants were identified by next-generation sequencing analysis (Figs 1e, S8, and  S9). These transgenic Carrizo citranges exhibited high editing efficiency (64.13%–100% for *CsLOB1* and 50%–100% for *CsWRKY22*), whereas transgenic Wanjincheng oranges showed low editing efficiency (23.27%–100% for *CsLOB1* and 22.81%–100% for *CsWRKY22*). The WO4 and CC2 lines had 100% editing frequency for both genes.

Canker resistance in all mutant plants was identified using the established inoculation protocol [2]. Compared with wild-type (WT) controls, LB1, LB2, and LB3 mutants exhibited a substantial reduction (88.63%–93.45%) in canker lesions, whereas WY3 and WY4 mutants showed 51.19% and 39.99% reduction at 10 days post-inoculation (dpi) (Fig. 1f and h). The canker resistance of WY1 and WY2 mutants did not change. For the double mutants of *CsLOB1* and *CsWRKY22*, canker lesions in WO2 and WO4 mutants were reduced by 66.78% and 54.77%, respectively (Fig. 1f and h). Notably, all five Carrizo citrange double mutants exhibited significant canker resistance, with lesion reductions ranging from 78.77% to 86.15% (Fig. 1g and i). *Xcc* growth was also significantly repressed in these mutants with increased disease resistance (Fig 1j–l).

Existing reports have demonstrated that editing the promoter (EBE_PthA4_) or the coding sequence of *CsLOB1* confers strong resistance to citrus canker [1, 2]. Similarly, the present study showed that editing the coding sequence of *CsLOB1* remarkably enhanced canker resistance. *CsLOB1* mutations in Wanjincheng orange conferred stronger resistance compared with *CsWRKY22* mutations (Fig. 1h), further underscoring the central role of *CsLOB1* in governing citrus resistance [1, 3]. Also, the double-mutant lines did not exhibit greater resistance to *Xcc* compared with *CsLOB1* single-mutant lines (Fig. 1h). This phenomenon was attributed to the lower mutation frequency of *CsLOB1* in double-mutant lines (Fig. 1e). Although the WO4 double-mutant line achieved 100% editing efficiency for both genes, it still developed larger lesions than *CsLOB1* single-mutant lines. The genotypic analysis revealed no substantial frameshift mutations in *CsLOB1* within the WO4 double-mutant line (Fig. S10). Thus, the enhanced resistance of this line primarily stemmed from *CsWRKY22* mutations. Furthermore, the double-mutant lines WO2 and WO3 displayed similar low editing efficiencies (~25% for both genes) (Fig. 1e), yet the WO2 line exhibited significant canker resistance (Fig. 1h). Previous studies have established that *Xcc* cells must breach the citrus epidermis to induce canker formation [4]. It has been indicated that *CsLOB1* mutations, specifically in epidermal cells, are sufficient to augment canker resistance [2]. Therefore, it is plausible that the high resistance of the WO2 line originates from gene mutations in epidermal cells. Additionally, all mutant plants displayed no significant difference in plant height (Figs S11a-S14a) or leaf phenotypes (Figs S11b-e to S14b-e) compared with WT plants when grown in a greenhouse. This finding was consistent with previous reports indicating that the modification of *CsLOB1* and *CsWRKY22* did not impair plant development [2, 5].

In summary, efficient simultaneous editing of *CsLOB1* and *CsWRKY22* in Wanjincheng orange and Carrizo citrange was achieved in this study by leveraging a *CRISPR*/Cas9 and PTG-mediated sgRNA processing system. The resulting mutants exhibited strong resistance to citrus canker, a highly destructive bacterial disease threatening the global citrus industry. The presented genome editing workflow also provides valuable references for multiplex gene editing in citrus.

## Supplementary Material

Web_Material_uhag137

## Data Availability

The data as well as materials and methods supporting this study are available in Supplementary materials file at online (https://pan.baidu.com/s/1sSWRW-pigT2DmXf847Vt0g?pwd=0000).

## References

[1] Zou X, Du M, Liu Y. et al. *CsLOB1* regulates susceptibility to citrus canker through promoting cell proliferation in citrus. Plant J. 2021;106:1039–57 33754403 10.1111/tpj.15217

[2] Peng A, Chen S, Lei T. et al. Engineering canker-resistant plants through CRISPR/Cas9-targeted editing of the susceptibility gene *CsLOB1* promoter in citrus. Plant Biotechnol J. 2017;15:1509–19 28371200 10.1111/pbi.12733 PMC5698050

[3] Long Q, Du M, Long J. et al. Transcription factor WRKY22 regulates canker susceptibility in sweet orange (*Citrus sinensis* Osbeck) by enhancing cell enlargement and *CsLOB1* expression. Hortic Res. 2021;8:50 33642585 10.1038/s41438-021-00486-2 PMC7917094

[4] Hu Y, Zhang JL, Jia HG. et al. *Lateral organ boundaries 1* is a disease susceptibility gene for citrus bacterial canker disease. Proc Natl Acad Sci USA. 2014;111:E521–9 24474801 10.1073/pnas.1313271111 PMC3910620

[5] Wang L, Chen S, Peng A. et al. CRISPR/Cas9-mediated editing of *CsWRKY22* reduces susceptibility to *Xanthomonas citri* subsp. *citri* in Wanjincheng orange (*Citrus sinensis* (L.) Osbeck). Plant Biotechnol Rep. 2019;13:501–10

