# Supplementary material for: Simultaneous editing of CsLOB1 and CsWRKY22 in citrus confers resistance to citrus canker
Source: Hortic Res. 2026 Apr 10;13(8):uhag137. doi: 10.1093/hr/uhag137 (PMC13446118; doi:10.1093/hr/uhag137)
Supplement: Supplementary file 1 [file web_material_uhag137.zip › Supplementary materials.docx]

**Simultaneous editing of *CsLOB1* and *CsWRKY22* in citrus confers resistance to citrus canker**

Mengyuan Hou ^1,†^, Rongrong Qu ^1, †^, Mengting He ^1^, Xiangling Chen^2^, Lanzhen Xu^1^, Juanjuan Ma^1^, Tiangang Lei^1^, Yongrui He^1^, Xiuping Zou^1,*^

^1^ Citrus Research Institute, Southwest University, Chongqing, People’s Republic of China

^2^ Horticultural Research Institute, Guangxi Academy of Agricultural Sciences, Chongqing, People’s Republic of China

^†^The authors contributed equally to this work.

* Corresponding author: E-mail: [zxping11@swu.edu.cn](mailto:zxping11@swu.edu.cn)

**Table S1. Sequence of synthetic genes used in this study**

| Gene  (Architecture) | Sequence (5’→3’) |
| --- | --- |
| AtU6-26::tRNA:gRNA scaffold-polyT in pATS plasmid | aagcttcgttgaacaacggaaactcgacttgccttccgcacaatacatcatttcttcttagctttttttcttcttcttcgttcatacagtttttttttgtttatcagcttacattttcttgaaccgtagctttcgttttcttctttttaactttccattcggagtttttgtatcttgtttcatagtttgtcccaggattagaatgattaggcatcgaaccttcaagaatttgattgaataaaacatcttcattcttaagatatgaagataatcttcaaaaggcccctgggaatctgaaagaagagaagcaggcccatttatatgggaaagaacaatagtatttcttatataggcccatttaagttgaaaacaatcttcaaaagtcccacatcgcttagataagaaaacgaagctgagtttatatacagctagagtcgaagtagtgattggaacaaagcaccagtggtctagtggtagaatagtaccctgccacggtacagacccgggttcgattcccggctggtgcacggtcttcgagaagacctgttttagagctagaaatagcaagttaaaataaggctagtccgttatcaacttgaaaaagtggcaccgagtcggtgcttttttt |
| gRNA scaffold:tRNA fusion in pGST plasmid | gttttagagctagaaatagcaagttaaaataaggctagtccgttatcaacttgaaaaagtggcaccgagtcggtgcaacaaagcaccagtggtctagtggtagaatagtaccctgccacggtacagacccgggttcgattcccggctggtgca |
| AtU6-26::gRNA fusion in pUC119-gRNA plasmid | aagcttcgttgaacaacggaaactcgacttgccttccgcacaatacatcatttcttcttagctttttttcttcttcttcgttcatacagtttttttttgtttatcagcttacattttcttgaaccgtagctttcgttttcttctttttaactttccattcggagtttttgtatcttgtttcatagtttgtcccaggattagaatgattaggcatcgaaccttcaagaatttgattgaataaaacatcttcattcttaagatatgaagataatcttcaaaaggcccctgggaatctgaaagaagagaagcaggcccatttatatgggaaagaacaatagtatttcttatataggcccatttaagttgaaaacaatcttcaaaagtcccacatcgcttagataagaaaacgaagctgagtttatatacagctagagtcgaagtagtgattgggtcttcgagaagacctgttttagagctagaaatagcaagttaaaataaggctagtccgttatcaacttgaaaaagtggcaccgagtcggtgcttttttt |

The sequences are annotated as follows:

AtU6-26 promoter: yellow background

pre-tRNA: green background

gRNA scaffold: turquoise background

Pol III terminator (TTTTTTT): pink background

**Table S2. Primers used in the study.**

| Primer name | Sequence (5'→3') | Comment |
| --- | --- | --- |
| LBsgRNAiv-11 | TTAATACGACTCACTATAGGAAGAACTTCAAATTACATGGGTTTTAGAGCTAGAAATAGCA | *In* *vitro* synthesis of *CsLOB1* gRNAs for *in vitro* cleavage analysis |
| LBsgRNAiv-12 | TTAATACGACTCACTATAGGTGATTAGGAGAAGGGAAGCGGTTTTAGAGCTAGAAATAGCA |  |
| LBsgRNAiv-13 | TTAATACGACTCACTATAGGGGAGAAGTAGAGAAAGTAGAGTTTTAGAGCTAGAAATAGCA |  |
| LBsgRNAiv-14 | TTAATACGACTCACTATAGGGGGCTAAGAACTATAGGCGGGTTTTAGAGCTAGAAATAGCA |  |
| LBsgRNAiv-15 | TTAATACGACTCACTATAGGCAAGGGCTAAGAACTATAGGGTTTTAGAGCTAGAAATAGCA |  |
| LBsgRNAiv-16 | TTAATACGACTCACTATAGGATTGTTGATGATTAGGAGAAGTTTTAGAGCTAGAAATAGCA |  |
| LBsgRNAiv-17 | TTAATACGACTCACTATAGGATTTCTCGACGCATCTGCGGGTTTTAGAGCTAGAAATAGCA |  |
| LBiv-f | ATTTGAAAGTACATCCATAACCCTG | Amplification of *CsLOB1* DNA templates for *in vitro* cleavage analysis |
| LBiv-r | CTCCAAAGTCTAATCACACAACTAA |  |
| LB1cs-13-f | ACTGAAGACCTGATTGGGAGAAGTAGAGAAAGTAGAGTTTTAGAGCTAGAAATAGC | Synthesis of *CsLOB1 PTG* using PCR |
| LB1cs-15-r | CCTATAGTTCTTAGCCCTTGTGCACCAGCCGGGAATCGAA |  |
| LB1cs-15-f | CAAGGGCTAAGAACTATAGGGTTTTAGAGCTAGAAATAGC |  |
| LB1cs-17-r | TGAGAAGACCCAAACCCGCAGATGCGTCGAGAAATTGCACCAGCCGGGAATCGAAC |  |
| LB1cs-f | ACTGAAGACCTGATTGGGAGAAGTA |  |
| LB1cs-r | TGAGAAGACCCAAACCCGCAGATGC |  |
| w22s-1-f | ACTGAAGACCTGATTGCCTCCTTTCGGTTGAGCTGCGTTTTAGAGCTAGAAATAGC | Synthesis of *CsWRKY22 PTG* using PCR |
| w22s-2-r | TAACAAAGTCGATGGAGGCCTGCACCAGCCGGGAATCGAA |  |
| w22s-2-f | GGCCTCCATCGACTTTGTTAGTTTTAGAGCTAGAAATAGC |  |
| w22s-3-r | TGAGAAGACCCAAACACTTATCACATGCGAGACAATGCACCAGCCGGGAATCGAAC |  |
| w22s-f | ACTGAAGACCTGATTGCCTCCTTTC |  |
| w22s-r | TGAGAAGACCCAAACACTTATCACA |  |
| Cas9-f | ATGGATTACAAGGATGATGATGATAAGGAT | Identification of transgenic plants using PCR |
| Cas9-r | TCTTCTCTCCTGGAAGCTGA |  |
| 3xLOBc1-f | GCAGCTCCTCCTCATCCCTT | Sanger sequencing of *CsLOB1* mutation |
| 3xLOBc1-r | ACAAAAGAAGTGCATACCTGCAA |  |
| 3xW22-f | GTCCGATCTTGACGAGGGAT | Sanger sequencing of *CsWRKY22* mutation |
| 3xW22-r | TTGAGCTGCTGCACCGATAAT |  |
| VM3 | GCATTTGATGACGCCATGAC | Quantification of *Xanthomonas citri* subsp. *Citri* in citrus |
| VM4 | TCCCTGATG CCTGGAGGATA |  |

Note: The red-highlighted sequences are gRNA spacers that guide Cas9 editing.

**Figure S1. Assembly of polycistronic *tRNA:gRNA* (*PTG*) gene using PCR** **and restriction digestion**. **(a)** Illustration of pATS and pGST plasmids. pGST contained a gRNA scaffold:tRNA fusion, which was employed as a template to synthesize each cistronic unit of *PTG* using PCR. pATS contained an expression cassette of AtU6-26::tRNA:sgRNA scaffold–polyT, which was used to construct the expression cassette of *PTG* driven by the *Arabidopsis* AtU6-26 promoter. polyT, RNA polymerase III terminator. **(b)** Schematic diagrams of *PTG* synthesis. Based on the gRNA scaffold:tRNA sequence in pGST, a series of specific primers were designed to generate the cistronic units of *PTG* containing various gRNA spacers at their termini by PCR amplification. The g_1_-f, g_2_-r, g_3_-r, …, g_n-1_-r, and g_n_-r primers had different gRNA spacers targeting different genomic sites and a sequence complementary to the 3’ end of the tRNA except for g_1_-f. The s_2_-f, s_3_-f, …, s_n-2_-f, and s_n-1_-f primers were complementary to the 5’ end of the sgRNA scaffold. The 4-bp core sequences of *Bbs*I sites (purple vertical lines indicating the cut by *Bbs*I) in the g_2_-r, g_3_-r, …, and g_n-1_-r primers were designed based on the four bases at the 3’ end of gRNA spacers to produce a complete gRNA without extra nucleotides. These core sequences were complementary to those in the s_2_-f, s_3_-f, …, s_n-2_-f, and s_n-1_-f primers. The g_1_-f and g_n_-r primers had no *Bbs*I sites. Following PCR amplification and *Bbs*I digestion, unique 4-bp overhangs were generated at the termini of each cistronic unit. These designed overhangs enabled the precise ligation of the units, thereby assembling a complete *PTG* with functional spacer sequences. Finally, the *PTG* was amplified from the ligated products using the p-f/p-r primers. The amplified product was further digested using *Bbs*I and inserted into the *Bbs*I-digested pATS vector to generate the *PTG* expression cassette controlled by the AtU6-26 promoter. AMP, Ampicillin resistance gene; TS, transcription start site; polyT, Pol III terminator; g_#_, gRNA spacers; Scaffold, gRNA scaffold.

**Figure S2.** **Validation of gRNA spacers for *CRISPR*/Cas9-mediated editing of *CsLOB1* in citrus.** (**a**) Schematic representation of the structure of *CsLOB1* and the positions of the candidate gRNA spacers (L11 to L17). PAM, protospacer adjacent motif. **(b)** *In* *vitro* cleavage analysis of the seven gRNA spacers. The DNA templates for this analysis were amplified from Wanjincheng oranges (*Citrus sinensis* Osbeck) using the primers LBiv-f/LBiv-r. Cleavage activity (%) of each gRNA spacer was estimated using ImageJ 2.0 software.

**Figure S3. Sequences of two alleles of *CsLOB1* and *CsWRKY22*.** Variant bases in green were used to name the alleles. Blue letters indicate other indels or single-nucleotide polymorphisms differing in two alleles of *CsLOB1*and *CsWRKY22*. Red letters indicate protospacer adjacent motif. The lines indicate the positions of the gRNA spacers used in this study. L# and W# represent gRNA spacer sequences designed to direct the editing of *CsLOB1* and *CsWRKY22*, respectively. pthA4 site, effector-binding element recognized by the PthA4 effector of *Xanthomonas citri* subsp.*. citri*.

**Figure S4. Identification of *PTG* transgenic Wanjincheng oranges (*Citrus sinensis* Osbeck) using GFP (a) and PCR (b and c) analyses.** An about 0.75-kb-long fragment of *Cas9* was amplified using Cas9-f/Cas9-r primers. M, DNA marker; P, pPTG_CsLOB1_ or pPTG_CsWRKY22_ vector; WT, wild-type control; LB# and WY#, *PTG_CsLOB1_* and *PTG_CsWRKY22_* transgenic plants, respectively. Scale bar = 1 cm.

**Figure S5. Sequences of *CsLOB1* mutations induced by *CRISPR*/Cas9 in Wanjincheng oranges (*Citrus sinensis* Osbeck).** The sequences of gRNA spacers are shown in red color, and the protospacer adjacent motif sites are shown in bold font. Red dashes indicate deleted nucleotides (d). The inserted (i) and substituted (s) nucleotides are highlighted in blue and yellow, respectively. “d#”, “i#”, and “s#” indicate the number of nucleotides deleted, inserted, and substituted, respectively. “× number” on the right-hand side indicates the number of detected clones with such a mutation. Frequency (%) was calculated based on the number of clones with mutations out of the total number of clones sequenced. LB#, *CsLOB1* mutants.

**Figure S6. Sequences of *CsWRKY22* mutations induced by *CRISPR*/Cas9 in Wanjincheng oranges (*Citrus sinensis* Osbeck).** The sequences of gRNA spacers are shown in red color, and the protospacer adjacent motif sites are shown in bold font. Red dashes indicate deleted nucleotides (d). The inserted (i) and substituted (s) nucleotides are highlighted in blue and yellow, respectively. Bold italic letters indicate natural alleles. “d#”, “i#”, and “s#” indicate the number of nucleotides deleted, inserted, and substituted, respectively. “× number” on the right-hand side indicates the number of detected clones with such a mutation. Frequency (%) was calculated based on the number of clones with mutations out of the total number of clones sequenced. WY#, *CsWRKY22* mutants.

**Figure S7. Identification of *PTG* transgenic plants using GFP (a) and PCR (b) analyses.** An about 0.75-kb-long fragment of *Cas9* was amplified using Cas9-f/Cas9-r primers**.** M, DNA marker; P, pPTG_L13+W3_ or pPTG_L17+W3_ vector; WT, wild-type control; CC#, *PTG_L17+W3_* transgenic Carrizo citranges (*Poncirus trifoliata* (L.) Raf. *× Citrus sinensis* Osbeck); WO1 to WO3, *PTG_L17+W3_* transgenic Wanjincheng oranges (*Citrus sinensis* Osbeck); WO4, *PTG_L13+W3_* transgenic Wanjincheng oranges. Scale bar = 1 cm.

**Figure S8. Genotypes of double mutants of *CsLOB1* and *CsWRKY22* in Wanjincheng oranges (*Citrus sinensis* Osbeck).** The sequences of gRNA spacers are shown in red color, and the protospacer adjacent motif sites are shown in bold font. Red dashes indicate deleted nucleotides (d). The inserted (i) nucleotides are highlighted in blue. “d#” and “i#” indicate the number of nucleotides deleted and inserted, respectively. “× number” on the right-hand side indicates the number of detected reads with such a mutation. Frequency (%) was calculated based on the number of reads with mutations out of the total number of reads sequenced. WO#, Wanjincheng orange mutants.

**Figure S9. Genotypes of double mutants of *CsLOB1* and *CsWRKY22* in Carrizo citrange (*Poncirus trifoliata* (L.) Raf. *× Citrus sinensis* Osbeck).** The sequences of gRNA spacers are shown in red color, and the protospacer adjacent motif sites are shown in bold font. Red dashes indicate deleted nucleotides (d). The inserted (i) nucleotides are highlighted in blue. “d#” and “i#” indicate the number of nucleotides deleted and inserted, respectively. “× number” on the right-hand side indicates the number of detected reads with such a mutation. Frequency (%) was calculated based on the number of reads or clones with mutations out of the total number of reads or clones sequenced. ^*^ indicates the data from Sanger sequencing. CC#, Carrizo citrange mutants.

**Figure S10. Comparison of the protein sequences of CsLOB1 between the WO4 mutant and wild-type (WT) control.** The orange rectangle indicates the mutations.

**Figure S11. Phenotypes of *CsLOB1* (LB) mutants growing for 4 years in the greenhouse. (a** and **b)** Plant **(a)** and leaf **(b)** shapes of LB mutants and wild-type (WT) control in the greenhouse. Scale bar = 1 cm. **(c–e)** Statistical analyses of leaf length **(c)**, leaf width **(d),** and leaf thickness **(e)**. Value is expressed as the mean ± standard deviation of 10 leaves per line. Letters above the bars indicate no significant differences compared with WT control (Duncan’s test, *P* > 0.05).

**Figure S12. Phenotypes of *CsWRKY22* (WY) mutants growing for 3 years in the greenhouse. (a** and **b)** Plant **(a)** and leaf **(b)** shapes of WY mutants and wild-type (WT) control in the greenhouse. Scale bar = 1 cm. **(c–e)** Statistical analyses of leaf length **(c)**, leaf width **(d),** and leaf thickness **(e)**. Value is expressed as the mean ± standard deviation of 10 leaves per line. Letters above the bars indicate no significant differences compared with WT control (Duncan’s test, *P* > 0.05).

**Figure S13. Phenotypes of *CsLOB1* and *CsWRKY22* double-mutant Wanjincheng oranges (WO)** **growing for 1 year in the greenhouse. (a** and **b)** Plant **(a)** and leaf **(b)** shapes of WO mutants and wild-type (WT) control in the greenhouse. Scale bar = 1 cm. **(c–e)** Statistical analyses of leaf length **(c)**, leaf width **(d),** and leaf thickness **(e)**. Value is expressed as the mean ± standard deviation of 10 leaves per line. Letters above the bars indicate no significant differences compared with WT control (Duncan’s test, *P* > 0.05).

**Figure S14. Phenotypes of *CsLOB1* and *CsWRKY22* double-mutant Carrizo citranges (CC)** **growing for 1 year in the greenhouse. (a** and **b)** Plant **(a)** and leaf **(b)** shapes of CC mutants and wild-type (WT) control in the greenhouse. Scale bar = 1 cm. **(c–e)** Statistical analyses of leaf length **(c)**, leaf width **(d),** and leaf thickness **(e)**. Value is expressed as the mean ± standard deviation of 10 leaves per line. Letters above the bars indicate no significant differences compared with WT control (Duncan’s test, *P* > 0.05).

**Materials and Methods**

**Plant and pathogen materials**

Plants used for citrus transformation were Wanjincheng orange (*Citrus sinensis* Osbeck) and Carrizo citrange (*Poncirus trifoliata* (L.) Raf. *× Citrus sinensis* Osbeck) planted in the National Center for Citrus Variety Improvement, Chongqing, China. All plant materials were grown in a greenhouse at 22℃–28℃. *Xanthomonas citri* subsp. *citri* (*Xcc*) strain was isolated from naturally infected citrus leaves from an orchard in Chongqing, China.

**Targeted** **gRNA spacer design and *in vitro* cleavage activity analysis**

The gRNA spacers targeting the coding sequence of *CsLOB1* were designed using the CRISPR-P Web tool ^1^. The gRNA spacers targeting the same sequences in the two alleles were selected for evaluation using *in vitro* cleavage activity analysis to completely edit two *CsLOB1* alleles in citrus. The *in vitro* cleavage activity analysis was performed as described previously ^2^. The specific sgRNAs (spacer:scaffold gRNAs) were synthesized using the E369_93-sgRNA Synthesis Kit (Novoprotein, Suzhou, China) following the manufacturer’s protocols. The DNA templates targeted by the gRNA spacers were amplified from the total genomic DNA of Wanjincheng oranges with the LBiv-f/LBiv-r primers (Table S2). The E365-E368_94-Cas9 Kit (Novoprotein, Suzhou, China) was used for *in vitro* cleavage activity analysis in a final volume of 20 µL, including 1 µg Cas9 protein, 100 nM sgRNA, 200 ng target DNA, and 10 µL of 2× Cas9 reaction buffer. Digestion was maintained at 37℃ for 1 h, and then the Cas9 protein was removed using proteinase. The digested products were separated on a 2% agarose gel. The DNA bands were quantified using ImageJ 2.0 (National Institutes of Health, MD, USA). The cleavage activity of the individual gRNA spacer was calculated using the proportion of digested products relative to the total amount of input target DNA.

***PTG* gene** **synthesis and vector construction**

The AtU6-26::tRNA:gRNA scaffold–polyT and gRNA scaffold:tRNA fusion frequencies (Table S1) were synthesized by Tsingke Biotechnology Co., Ltd. (Beijing, China) to construct *PTG* gene and finally inserted into pUC57 vectors to generate the pATS and pGST plasmids (Fig. S1a), respectively. The *PTG* genes for editing *CsLOB1* and *CsWRKY22* were generated as described in Figure S1b and then inserted into the pATS plasmid using *Bbs*I. Then, the *PTG* cassettes were digested using *Sal*I/*Bam*HI and inserted into the *Sal*I/*Bam*HI-cut pNmG-mCas9 vector (Fig. 1c) for citrus transformation.

**Citrus transformation**

Transgenic plants were generated by the *Agrobacteria tumefasciens*–mediated transformation of citrus epicotyl segments following a conventional protocol ^3^. Transgenic shoots were identified using GFP fluorescence, and then micrografted onto “Ziyang xiangcheng” (*Citrus* × *junos Siebold* ex Tanaka) seedlings. After 1 month, recovered transgenic plants were grafted onto “Ziyang xiangcheng” rootstock in the greenhouse and further examined using PCR. The presence of transgenes in transgenic plants was confirmed by PCR using the primer pair cas9-f/cas9-r (Table S2).

**Sequencing analysis of mutations in transgenic plants**

Sanger sequencing and next-generation sequencing (NGS) analysis were used to investigate mutation genotypes and editing frequency in transgenic plants. For Sanger sequencing analysis, an about 500-bp sequence containing target sites was amplified from transgenic plants and cloned into the pGEM-T Easy vector (Promega, WI, USA). A minimum of 20 clones per transgenic plant were sequenced. The NGS analysis of mutations was performed using an Hi-TOM sequencing platform ^4^. Next, 150- to 300-bp sequences across target sites were amplified from transgenic plants using primers containing specific bridging sequences. The PCR products were then sequenced and analyzed using the Hi-TOM sequencing platform at the China National Rice Research Institute, Zhejiang, China. More than 3000 paired-end reads were generated per amplicon.

**Assay of citrus canker resistance in transgenic plants**

Using WT plants as controls, citrus canker resistance in mutant plants was evaluated following the established inoculation protocol ^5^. Three mature leaves per transgenic plant including WT controls were examined. Six infection sites, each comprising six needle pricks, were created on each leaf using an inoculation needle (0.5 mm in diameter). A 1-µL *Xcc* suspension (1×10^8^ CFU/mL) was dropped onto each prick. The inoculation was maintained in an incubator with a 16-h photoperiod and 80% relative humidity at 28℃. At 7 dpi, the symptoms were photographed, and the lesion area was measured using ImageJ 2.0 software.

A quantitative polymerase chain reaction (qPCR) method ^6^ was used to determine *Xcc* growth in transgenic plants. Leaf disks (0.25 cm^2^) from three inoculated sites were pooled for DNA extraction. The abundance of the *pthA* gene of *Xcc* in the extracted DNA samples was then quantified by qPCR using the VM3 and VM4 primers (Table S2). Based on the contents of the *pthA* gene, the *Xcc* population (*Xcc* cells/µg of citrus DNA) in infected leaves was estimated using the following formula: *Xcc* cells/µg of citrus DNA = 10 × [(38.3 – Ct)/3.56] ^6^. The experiments were conducted in three independent biological replicates.

**References**

1.	Lei, Y., Lu, L., Liu, H.Y. *et al.* CRISPR-P: a web tool for synthetic single-guide RNA design of CRISPR-system in plants*.* *Mol Plant*. 2014;**7**(9):1494-1496

2.	Wang, L., Chen, S., Peng, A. *et al.* CRISPR/Cas9-mediated editing of *CsWRKY22* reduces susceptibility to *Xanthomonas citri* subsp. *citri* in Wanjincheng orange (*Citrus sinensis* (L.) Osbeck)*.* *Plant Biotechnol Rep*. 2019;**13**(5):501-510

3.	Peng, A., Xu, L., He, Y. *et al.* Efficient production of marker-free transgenic ‘Tarocco’ blood orange (*Citrus sinensis* Osbeck) with enhanced resistance to citrus canker using a Cre/loxP site-recombination system*.* *Plant Cell Tiss Org (PCTOC)*. 2015;**123**(1):1-13

4.	Liu, Q., Wang, C., Jiao, X. *et al.* Hi-TOM: a platform for high-throughput tracking of mutations induced by CRISPR/Cas systems*.* *Sci China Life Sci*. 2019;**62**(1):1-7

5.	Peng, A., Chen, S., Lei, T. *et al.* Engineering canker-resistant plants through CRISPR/Cas9-targeted editing of the susceptibility gene *CsLOB1* promoter in citrus*.* *Plant Biotechnol J*. 2017;**15**(12):1509-1519

6.	Stover, E.W. & McCollum, T.G. Levels of *Candidatus* Liberibacter asiaticus and *Xanthomonas citri* in diverse *citrus* genotypes and relevance to potential transmission from pollinations*.* *HortScience*. 2011;**46**(6):854-857
